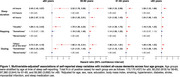# Differential associations of self‐reported nighttime and daytime sleep habits with incident dementia across age groups from midlife to late life

**DOI:** 10.1002/alz70860_107232

**Published:** 2025-12-23

**Authors:** Sasha Milton, Jiahe Wei, Haizhen Chen, Kristine Yaffe, Yi Fang, Xiao Tan, Yue Leng

**Affiliations:** ^1^ University of California, San Francisco, San Francisco, CA, USA; ^2^ Zhejiang University, Hangzhou, Zhejiang, China; ^3^ San Francisco Veterans Affairs Health Care System, San Francisco, CA, USA; ^4^ Uppsala University, Uppsala, Uppsala, Sweden

## Abstract

**Background:**

Growing evidence indicates that sleep disturbances are associated with an increased risk of dementia. However, most studies have focused on older adults, raising concerns about reverse causality. Investigating whether the relationship between sleep and dementia differs from midlife to late life is crucial for disentangling its directionality.

**Method:**

We examined 410,162 UK Biobank participants (median [range] age=58 [38‐73] years; 55.0% female; 8.1% non‐White) without dementia who self‐reported 24‐hour sleep duration, napping, and unintentional daytime dozing. We used Cox models to evaluate associations between sleep variables and incident all‐cause dementia (ACD, determined via International Classification of Diseases codes) in four age groups (≤55, 56‐60, 61‐65, >65 years at the sleep visit) and tested for sleep‐age interactions.

**Result:**

Over a median (interquartile range) follow‐up of 13.6 (12.9‐14.3) years, there were 7,592 (1.9%) incident ACD cases, including 437 (0.25%), 819 (1.0%), 2,644 (2.7%), and 3,692 (6.2%) cases in the ≤55, 56‐60, 61‐65, and >65 groups, respectively. After adjustment for demographics, comorbidities, and sleep medication use, participants with sleep duration <6 hours or >9 hours showed increased ACD risk relative to participants with 6‐9 hour sleep duration across all age groups, with particularly strong effect sizes in the ≤55 group (hazard ratio, HR [95% confidence interval, CI] for <6 hours: 2.28 [1.67,3.12]; >9 hours: 2.40 [1.45,3.97]) and the 56‐60 group (HR [95% CI] for <6 hours: 1.55 [1.20,2.01]; >9 hours: 2.52 [1.78,3.56]). Similarly, there were particularly strong associations of “usually” napping (versus “never/rarely”) and dozing “often/all of the time” (versus “never/rarely”) with ACD in the ≥55 group (HR [95% CI] for napping: 1.68 [1.12,2.53]; dozing: 2.72 [1.81,4.10]) and 56‐60 group (HR [95% CI] for napping: 1.70 [1.30,2.24]; dozing: 1.66 [1.19,2.31]). All sleep‐age interactions were significant (*p* <0.00001).

**Conclusion:**

Participants with short or long sleep duration, as well as those who napped or dozed frequently, exhibited approximately doubled dementia risk, particularly among individuals aged 60 years and younger. Sleep patterns during midlife are critical for dementia risk, and sleep disturbances likely precede dementia onset.